# P-1936. Does SARS-CoV-2 Infection or Vaccination Impact Menstrual Health? Results From a Longitudinal Cohort Study Among Military Health System Beneficiaries

**DOI:** 10.1093/ofid/ofae631.2095

**Published:** 2025-01-29

**Authors:** Caleb Mayes, Catherine Berjohn, Celia Byrne, Rhonda E Colombo, Evan Ewers, Ryan Flanagan, Anthony C Fries, Anuradha Ganesan, Milissa Jones, Tahaniyat Lalani, Derek Larson, David A Lindholm, Katrin Mende, Rupal Mody, Carlos Maldonado, Ryan C Maves, Jennifer Rusiecki, David Saunders, Christina Schofield, Robert O’Connell, Mark Simons, David R Tribble, Brian Agan, Timothy Burgess, Simon Pollett, Stephanie A Richard

**Affiliations:** USUHS, Bethesda, Maryland; Naval Medical Center San Diego, San Diego, California; Uniformed Services University of the Health Sciences, Bethesda, Maryland; Infectious Disease Clinical Research Program, USUHS; Henry M. Jackson Foundation for the Advancement of Military Medicine, Inc., Bethesda, Maryland; Fort Belvoir Community Hospital, Fort Belvoir, Virginia; Tripler Army Medical Center, Honolulu, Hawaii; U.S. Air Force School of Aerospace Medicine, Dayton, Ohio; Infectious Disease Clinical Research Program, USUHS; Henry M. Jackson Foundation for the Advancement of Military Medicine Inc, Bethesda, Maryland; Uniformed Services University of the Health Sciences, Bethesda, Maryland; Naval Medical Center Portsmouth, Portsmouth, Virginia; Fort Belvoir Community Hospital and Uniformed Services University, Fort Belvoir, Virginia; Department of Medicine, Uniformed Services University of the Health Sciences; Brooke Army Medical Center, San Antonio, TX; Infectious Disease Clincial Research Program, JBSA Ft Sam Houston, Texas; William Beaumont Army Medical Center, El Paso, Texas; Womack Army Medical Center, Fort Bragg, North Carolina; Wake Forest University School of Medicine, Winston-Salem, North Carolina; Uniformed Services University of the Health Sciences, Bethesda, Maryland; Uniformed Services University of the Health Sciences, Bethesda, MD, USA, Bethesda, Maryland; Madigan Army Medical Center, Tacoma, Washington; Infectious Disease Clinical Research Program, USUHS, Bethesda, Maryland; IDCRP, Bethesda, Maryland; Uniformed Services University of the Health Sciences, Bethesda, Maryland; Infectious Disease Clinical Research Program, Department of Preventive Medicine and Biostatistics, Uniformed Services University of the Health Sciences, Bethesda, MD, USA, Bethesda, Maryland; Infectious Disease Clinical Research Program, Department of Preventive Medicine and Biostatistics, Uniformed Services University of the Health Sciences, Bethesda, MD, USA, Bethesda, Maryland; Infectious Disease Clinical Research Program, Department of Preventive Medicine and Biostatistics, Uniformed Services University of the Health Sciences, Bethesda, MD, USA, Bethesda, Maryland; Infectious Disease Clinical Research Program, Department of Preventive Medicine and Biostatistics, Uniformed Services University of the Health Sciences, Bethesda, MD, USA, Bethesda, Maryland

## Abstract

**Background:**

The impact of SARS-CoV-2 infection and vaccination on menstruation is unclear. The goal of this study is to quantify the relationships between history of SARS-CoV-2 vaccination and infection and incident, clinically documented menstrual irregularities using data from the EPICC study in Military Health System (MHS) beneficiaries.

**Figure 1.**
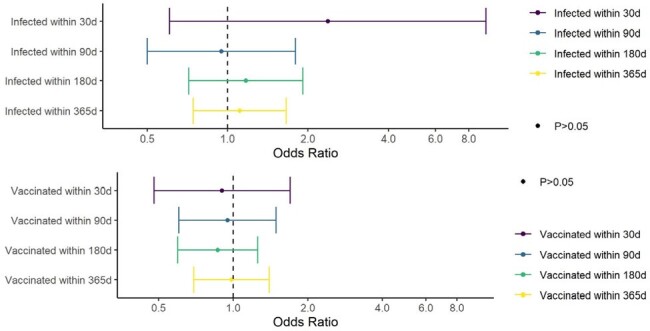
Odds of menstrual irregularities associated with having been infected with or vaccinated against SARS-CoV-2 in certain timeframes (30, 90, 180, and 365 days prior to diagnosis or matched timepoint), after controlling for comorbidities in cases and controls matched on race/ethnicity, five-year age group, and BMI category in the Epidemiology, Immunology, and Clinical Characteristics of emerging infectious diseases with pandemic potential (EPICC) cohort.

**Methods:**

This study included 18–50-year-old women who did not have pre-specified characteristics (e.g., pregnancy, breastfeeding, menopause) or pre-existing diagnoses of menstrual irregularities prior to the pandemic in their electronic medical record (MHS Data Repository (MDR)). Cases had a new diagnosis of a menstrual irregularity in the MDR between 3/2020 and 12/2022 and were matched 1:1 with a control of the same race/ethnicity, BMI category, and age category who did not have a menstrual irregularity diagnosis during that same time. Proportions with/without a history of SARS-CoV-2 infection and/or vaccination were compared. A logistic regression model provided estimates of the associations between SARS-CoV-2 infection or vaccination (in 30-, 90-, 180- or 365-days prior to diagnosis or matched timepoint) and diagnosis of menstrual irregularities.

**Table 1.**
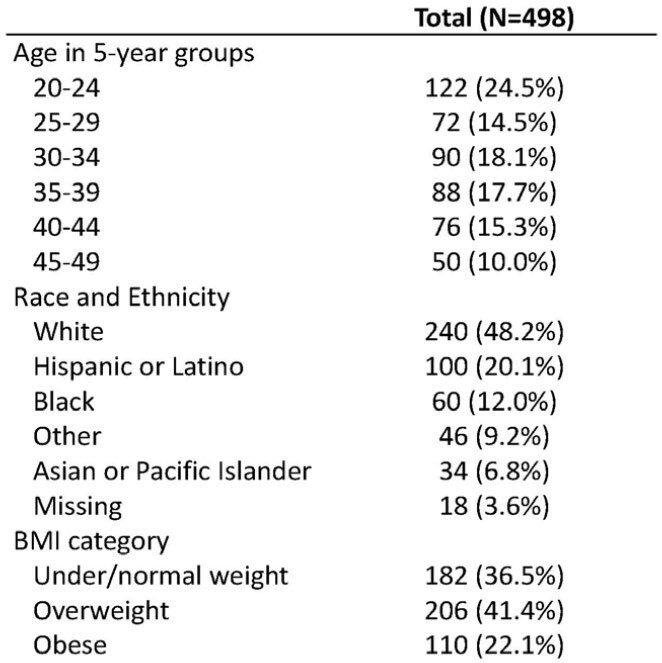
Matched characteristics in those with and without menstrual irregularities in EPICC (N=249 cases, 249 controls)

**Results:**

There were 270 participants (out of 1,156 women) with incident clinically documented menstrual irregularities identified over 2.8 years (8.3% per year from 3/20 to 12/22). Based on individual matching, 249 cases and controls were included in analyses (N=498, 21 cases did not have a matched control and were dropped) (Tables 1 & 2). Fewer controls than cases had a positive SARS-CoV-2 test in the prior 30, 180, and 365 days, whereas more controls reported being vaccinated in all time periods, but these were not statistically different (p >0.2) (Table 3). No statistically significant associations between 30, 90, 180, or 365-day history of SARS-CoV-2 infection or vaccination and menstrual irregularities were identified (Figure 1).

**Table 2.**
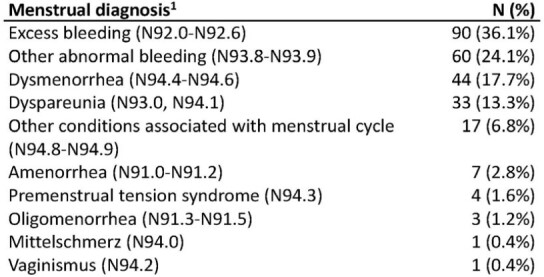
Incident menstrual-associated irregularities reported in 249 EPICC participants 1 - 11 (4.4%) of the participants had more than one diagnosis

**Conclusion:**

No relationship between recent SARS-CoV-2 infection/vaccination and clinically attended menstrual irregularities was observed in this analysis, but further studies are needed to confirm these findings. Future work will include analyses based on each vaccination and infection event using Cox proportional hazards models.

**Table 3.**
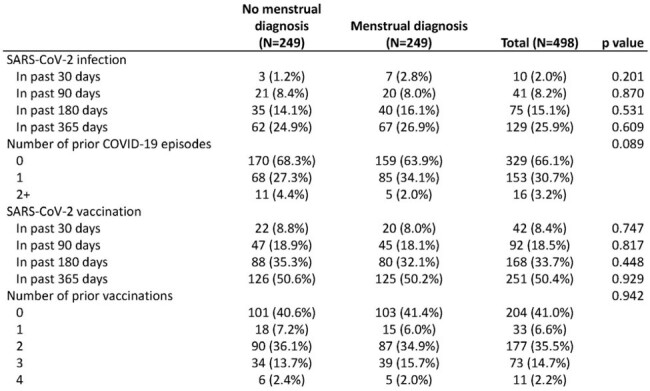
Participants’ history of SARS-CoV-2 infection and vaccination among those with and without menstrual irregularities in EPICC. P-values generated using Pearson’s Chi-squared tests.

**Disclosures:**

Ryan C. Maves, MD, AiCuris: Grant/Research Support|Biotest: Grant/Research Support|GeoVax: Grant/Research Support|Shionogi: Advisor/Consultant|Shionogi: Honoraria|Sound Pharmaceuticals: Grant/Research Support Mark Simons, PhD, Astrazeneca: Grant/Research Support Timothy Burgess, MD, MPH, AstraZeneca: The IDCRP and HJF were funded to conduct an unrelated phase III COVID-19 monoclonal antibody immunoprophylaxis trial as part of US Govt COVID Response Simon Pollett, MBBS, AstraZeneca: The IDCRP and HJF were funded to conduct an unrelated phase III COVID-19 monoclonal antibody immunoprophylaxis trial as part of US Govt COVID Response

